# Role of Branched‐Chain Amino Acids in Mitigating Osteosarcopenia: An Experimental Study Using Ovariectomised Mice Models

**DOI:** 10.1002/jcsm.70105

**Published:** 2025-10-29

**Authors:** Geum‐Hwa Lee, Hwa‐Young Lee, Young‐Jae Lim, Se‐Woong Ko, Han‐Jung Chae, Sun‐Jung Yoon

**Affiliations:** ^1^ Research Institute of Clinical Medicine of Jeonbuk National University‐Biomedical Research Institute of Jeonbuk National University Hospital Jeonju Republic of Korea; ^2^ Non‐Clinical Evaluation Center, Biomedical Research Institute Jeonbuk National University Hospital Jeonju Republic of Korea; ^3^ School of Pharmacy and Institute of New Drug Development Jeonbuk National University Jeonju Republic of Korea; ^4^ Department of Orthopedic Surgery Jeonbuk National University Medical School Jeonju Republic of Korea

**Keywords:** branched‐chain amino acids (BCAA), muscle atrophy, osteosarcopenia, postmenopausal sarcopenia and osteoporosis, sclerostin and Wnt signalling

## Abstract

**Background:**

Osteosarcopenia, characterised by concurrent bone loss and muscle atrophy, presents a significant challenge in aging populations, particularly in postmenopausal women. The current therapeutic options potentially treat bone and muscle loss independently, highlighting the importance of an integrated approach. This study aimed to investigate the effects of branched‐chain amino acid (BCAA) supplementation on muscle and bone health using ovariectomised (OVX) mice, a model for postmenopausal osteoporosis and sarcopenia.

**Methods:**

Female C57BL/6 mice were divided into sham‐operated and OVX groups, with OVX mice further subdivided to receive 0.25 mg/kg (Low) or 1 mg/kg (High) of BCAA supplementation for 16 weeks. Muscle mass, function and mitochondrial health were assessed alongside bone mineral density (BMD), bone turnover markers and histological evaluations. Additionally, the study explored mechanistic insights into sclerostin modulation and its influence on Wnt signalling through plasma and tissue analyses.

**Results:**

The hind limb fat mass was increased in the OVX group but reduced with BCAA supplementation, while hindlimb lean mass (*p* < 0.01) and total lean mass (*p* < 0.001) were significantly higher in the OVX + High‐BCAA group compared with the OVX group. Gastrocnemius muscle weight was lower in the OVX group but improved (*p* < 0.05) with both Low‐ and High‐BCAA supplementation. BCAA preserved bone microarchitecture by improving cortical thickness (*p* < 0.01) and modulating bone turnover markers, including osteocalcin (*p* < 0.01) levels. Plasma sclerostin levels were regulated, suggesting a role in bone remodelling. In muscle, BCAA enhanced hypertrophy by upregulating MHC expression (*p* < 0.05) and downregulating atrophy markers such as Atrogin‐1 (Low‐BCAA, *p* < 0.001; High BCAA, *p* < 0.001) and MuRF‐1 (Low‐BCAA, *p* < 0.01; High BCAA, *p* < 0.001). Additionally, BCAA mitigated the cytotoxic effects of H_2_O_2_ in osteocytic MLO‐Y4 cells, reducing sclerostin levels (*p* < 0.05) and improving cellular viability (*p* < 0.05). In C2C12 cells, BCAA reversed sclerostin‐induced muscle atrophy (*p* < 0.01), increasing MHC expression (*p* < 0.01) and myotube diameter (*p* < 0.01) while reducing Atrogin‐1 (*p* < 0.01) and MuRF‐1 (*p* < 0.001) expression.

**Conclusions:**

BCAA supplementation alleviates muscle atrophy and partially preserves bone microarchitecture in OVX mice. Importantly, our data highlight bone‐derived sclerostin as a molecular link that transmits bone signals to muscle; BCAA mitigates osteosarcopenia by modulating this bone‐to‐muscle endocrine axis via Wnt signalling. Although improvements in bone structure were modest, the findings position BCAAs as a promising adjunct therapy targeting the integrated bone–muscle unit.

## 1 Introduction

Clinically, osteosarcopenia is a co‐occurrence of osteoporosis and sarcopenia, especially in the elderly population, which underscores its significant impact on individual health. Sarcopenia is highly prevalent, particularly in individuals with severe osteoporosis. These observations demonstrate an intricate interaction between bone loss and deteriorating muscle health with advancing age. Thus, understanding this complex nature of muscle‐bone interactions is crucial for identifying the compounded risk factors and mechanisms underlying sarcopenia, which significantly influence the quality of life and morbidity in the aging population [1, 2]. This bone‐muscle cross‐talk has spurred interest in research into the interrelations between sarcopenia and osteoporosis [3, 4, 5, 6]. These investigations aim to uncover potential therapeutic targets and approaches that could lead to comprehensive treatment against muscle and bone deterioration. One such pathway involves sclerostin, a key osteocyte‐derived protein known to inhibit Wnt signalling, which negatively regulates bone formation [7]. Specifically, sclerostin expression is downregulated by mechanical loading, a process that promotes bone formation, whereas conditions such as physical inactivity, aging and osteoporosis are associated with elevated sclerostin levels, contributing to bone loss [8, 9]. Importantly, sclerostin also plays a role in muscle‐bone signalling, where muscle function and mass changes can modulate bone metabolism. This interaction is critical in understanding the pathophysiology of osteosarcopenia and identifying novel therapeutic targets for its treatment. Therefore, targeting sclerostin offers a promising way to enhance muscle and bone health, delivering a dual benefit in aging populations.

Recent reports suggest that branched‐chain amino acids (BCAAs) significantly ameliorate sarcopenia by promoting muscle protein synthesis and enhancing muscle mass [10, 11, 12]. However, the effects of BCAAs on bone health, especially in postmenopausal sarcopenia and osteoporosis, have been less explored. Given the critical role of muscle‐bone cross‐talk in osteosarcopenia, there is growing interest in the potential of BCAAs to modulate both muscle and bone health. Interestingly, BCAAs influence on muscle mass and function could indirectly impact bone metabolism through pathways involving myokines and osteokines, including sclerostin. Thus, understanding these mechanistic components potentially leads to new avenues in therapeutic strategies for addressing the dual challenge of muscle and bone loss. This study aims to investigate the effects of BCAA supplementation on muscle and bone health in an ovariectomised (OVX) mouse model of postmenopausal osteoporosis. The OVX model closely mimics the hormonal changes observed in postmenopausal women, making it an ideal system for studying the impact of BCAAs on bone and muscle cross‐talk. Further, the investigation aims to explore potential synergistic effects between muscle and bone systems, which help combat osteosarcopenia.

## 2 Methods

### 2.1 Animals and Experimental Groups

Female (7 weeks‐old) C57/BL6 mice were purchased from Orient Science Co. (Seongnam, Korea). Mice were housed at 22 ± 2°C with a 12‐h light/day cycle under 55%–60% humidity in the SPF facility. Animals were provided with a standard chow diet with free access to water. Mice were acclimatised to the experimental conditions under controlled conditions for 1 week prior to the start of the study. Following 7 days of acclimatisation, the animals were randomly allocated to five groups of 10 animals. Two groups were sham‐operated (Sham), while the other three groups underwent bilateral OVX to induce osteosarcopenia as previously described [13, 14]. After surgery under ketamine anaesthesia, mice were fostered for 2 weeks to allow for recovery and removal of endogenous sex hormones. To examine the effects of BCAA on osteosarcopenia, BCAA (LIVACT, a brand name of BCAA granules that consist of 46% leucine, 28% valine and 23% isoleucine, SAMIL, Seoul, Korea) or vehicle (water, 5 μL/g of body weight) was administered by daily intragastric gavage for 16 weeks. The study groups include Vehicle, Sham‐operated mice; BCAA, Sham‐operated mice administered with 1 mg/g/day BCAA; OVX, OVX mice treated with vehicle; Low, OVX mice administered with 0.25 mg/g of body weight/day BCAA; High, OVX mice administered with 1 mg/g of body weight/day BCAA (*n* = 8 animals/group). The body weight of all experimental mice was assessed once a week during the intervention period. After 16 weeks of intervention, all animals were sacrificed by ketamine anaesthesia. Blood samples were collected for ELISA tests, and femurs were collected for micro‐CT scanning and histological examination, Gastrocnemius muscles were dissected to measure volume and weight. For body composition analysis, data from two animals were excluded due to technical issues during tissue collection or measurement. Thus, the final sample size for fat mass evaluation was *n* = 8 per group. All the animal procedures were performed strictly following the Jeonbuk National University Hospital Institutional Animal Care and Use Committee's guidelines for the care and use of laboratory animals (JBUH‐IACUC‐2022‐25).

### 2.2 Dual‐Energy X‐Ray Absorptiometry (DEXA)

Whole‐body, hindlimb and forelimb lean mass, fat mass and bone mineral density (BMD) from lumbar vertebrae and femurs were measured by small animal dual‐energy X‐ray absorptiometry (DEXA; iNSiGHT VET *DXA*, OsteoSys, Seoul, Korea) to assess the efficacy of the model.

### 2.3 Skeletal Muscle Atrophy

The left gastrocnemius muscles were fixed, demineralised, embedded in paraffin and appropriately sectioned. Then, sections were deparaffinised with xylene, rehydrated in gradient alcohol and stained with haematoxylin and eosin (H&E). The muscle histology was observed under a light microscope (EVOS M7000, Thermo Fisher Scientific, USA). Each cross‐sectional area (CSA) of 100 muscle fibres was measured using the ImageJ software (National Institutes of Health, Stapleton, NY, USA).

### 2.4 Grip Strength Analysis

The maximal muscle strength of the mice was determined by measuring grip strength at 16 weeks using a grip strength meter (Bioseb, Chaville, France). At the end of the oral administration period, mice were placed with their forelimbs or all limbs on a grid, and their grip strength was immediately measured before they fell from the bar. Each animal's mean of five measurements was calculated. Skeletal muscle strength was expressed in grams.

### 2.5 Immunohistological Analysis of Muscle Tissues

The frozen excised gastrocnemius muscle tissues were cut centrally along the width and embedded in Tissue‐Tek O.C.T. Compound (Sakura Finetek Japan Co. Ltd., Tokyo, Japan). Frozen sections sliced to a thickness of 10 μm using a cryostat (HM 550, Thermo Fisher Scientific, USA). Double‐colour immunofluorescent staining was performed to evaluate the diameter of each type I and II fibre as described previously [15]. Briefly, primary antibodies against MHCI (1:200, BA‐F8, DSHB), MHCIIa (1:500; SC‐71, DSHB) and MHCIIb (1:100; BF‐F3, DSHB) were applied to tissue sections and incubated overnight at 4°C. After washing three times with phosphate‐buffered saline (PBS), the sections were incubated with the following fluorescent‐labelled secondary antibodies at room temperature for 1 h: Alexa Flour 568‐labelled goat anti‐mouse IgG1 (1: 500; Invitrogen Corporation, Carlsbad, CA, USA, A‐11004), Alexa Flour 488‐labelled goat anti‐mouse IgG2b (1:  500; Invitrogen Corporation, A‐21141) and Alexa Flour 488‐labelled goat anti‐rabbit IgG (H + L) (1:  500; Invitrogen Corporation, A‐11008). For quantification of the CSA of the muscle fibres, the CSA of the specimens were evaluated using 300 fibres of type IIb muscle fibres or 100 fibres of Type I muscle fibres at 100 × magnification per animal with Image J analysis (NIH).

### 2.6 Cell Culture

For studies involving BCAA‐reduced/free or amino acid‐free media, amino acid‐free DMEM was prepared and supplemented with BCAA, 10% foetal bovine serum (FBS) and 1% penicillin/streptomycin (P/S). Custom BCAA‐free/amino acid‐free DMEM was provided by Invitrogen. The C2C12 cells were incubated in a 5% CO_2_ incubator at 37°C. When cells reached 100% confluency, the medium was changed to a differentiation medium [DM, DMEM with 2% Horse Serum (HS), 1% P/S] every 2 days, and cells were left to differentiate for 4 days fully. For the myotube atrophy study induced by TNF‐α, C2C12 cells were induced to differentiate in DM for 3 days and treated with TNF‐α and the indicated concentration of 0.25, 0.5, and 1 mM BCAA, followed by incubation in DM for 1 day. C2C12 cells were plated in six‐well plates at a density of 1.0 × 10^5^ cells per well and allowed to attach and grow overnight. Cells differentiated into myotubes for 6 days in DM without sclerostin treatment were used as a negative control. C2C12 myotubes were treated with 100 ng/mL sclerostin and 0.25, 0.5 or 1 mM BCAA in DM for 48 h. MLO‐Y4, a murine long bone‐derived osteocyte‐like cell line (referred to hereafter as MLO‐Y4 cells), was cultured as described [16]. Briefly, cells were seeded on the Type I collagen‐coated plates and cultured in α‐MEM supplemented with 2.5% FBS, 2.5% Bovine Calf Serum (BCS), and 1% P/S. Cells were maintained at approximately 60% confluence throughout the culture period. After exposure to 100 μM H_2_O_2_ for 6 h, cells were treated with the indicated concentrations of 0.25, 0.5 and 1 mM BCAA.

### 2.7 Measurements of Myotube Diameter

Myotube diameter was measured as described earlier with minor modifications [17]. Images of myotubes were visualised at ×20 magnification using fluorescent microscopy (EVOS M5000, Thermo Fisher Scientific). The myotube diameter was measured for at least 100 myotubes in each group (five random fields per well, three wells per experiment, repeated at least three times) using the Image J analysis (NIH). The average diameter per myotube was determined by calculating the mean of 10 short‐axis measurements taken along the length of the myotube.

### 2.8 Statistical Analysis

Each experimental group initially consisted of 10 mice (*n* = 10). Due to occasional technical challenges during sample processing (e.g., tissue damage or insufficient material), some assays were conducted on a reduced number of animals. Analyses were performed using available valid samples, and for cases involving fewer than 10 mice, animals were selected without bias from the original groups. The exact number of animals included in each experiment is indicated in the corresponding figure legends. Statistical analyses were conducted using appropriate tests based on the actual sample size for each dataset.

**FIGURE 1:**
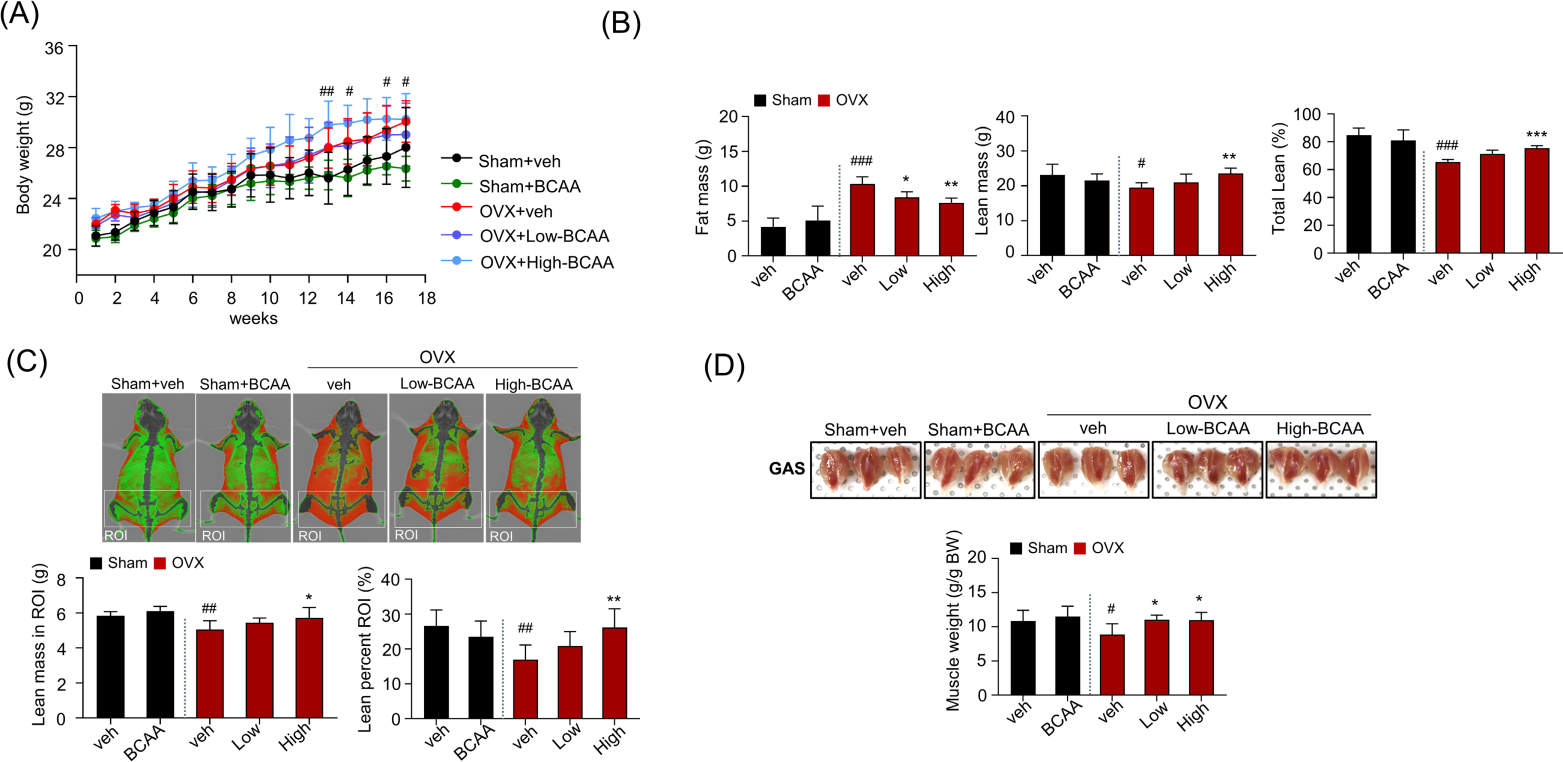
Effects of BCAA on osteosarcopenia in ovariectomized mice. (A) Changes in body weight during the experimental period. (B) Whole‐body composition using DEXA, including fat mass and mean mass. (C) Representative DEXA images of skeletal muscle (top) and lean percentage in ROIs (bottom). (D) Representative images of the GAS muscles taken at the end of the investigation (top). The respective masses of the GAS muscles (bottom) determined by normalising with most recently measured body weight (g/g). In panel (A), symbols represent significance between Sham + veh and OVX + veh only. Data are presented as mean ± SD (*n* = 10 (A), *n* = 8 (B) to (D) animals/group). (#*p* < 0.05, ##*p* < 0.01, ###*p* < 0.001 vs. Sham+veh; *p < 0.05, **p < 0.01, ***p < 0.001 vs. OVX + veh). OVX, ovariectomy; BCAA, *a branched*‐chain amino acid. BCAA, Sham‐operated mice administered with 1 mg/g/day BCAA; DEXA, dual‐energy X‐ray absorptiometry; GAS, gastrocnemius; High, OVX mice administered with 1 mg/g of body weight/day BCAA; Low, OVX mice administered with 0.25 mg/g of body weight/day BCAA; OVX, OVX mice treated with vehicle; Veh, Sham‐operated mice.

All statistical calculations and relevant analyses were performed using the GraphPad Prism version 10.0 (GraphPad Software, San Diego, CA, USA). Figure 1A was analysed using two‐way repeated‐measures analysis of variance (ANOVA) with group (between‐subject factor) and time (within‐subject factor) as variables. When significant interactions were detected, Tukey's multiple comparisons test was applied as a post hoc analysis. All other datasets were analysed using one‐way ANOVA followed by Tukey's post hoc test. Data are shown as the mean ± SD *p* < 0.05 was considered statistically significant.

## 3 Results

### 3.1 Body Weight and Lean Body Mass

Body weight and body composition did not differ among the groups at baseline. Body weight increased throughout the experiments, but an increment of body weight was more pronounced in OVX groups, with or without BCAA administration, beginning at 10 weeks post‐surgery (Figure 1A). The whole body, hindlimb, forelimb lean mass, fat mass and BMD from lumbar vertebrae and femurs were measured by DEXA every 4 weeks. Whole‐body lean mass in the Sham group remained stable at 4, 8, 12 and 16 weeks (Figure S1A), whereas a decline was observed in the OVX group without BCAA after 16 weeks. Fat mass of the hindlimb was higher in the OVX group compared with the Sham group, which was lower in the OVX + BCAA intake group (Figure 1B). Further, BCAA intake increased whole‐body lean mass in the OVX group after 16 weeks. Lean mass of hindlimb and total lean mass in OVX + high‐BCAA was higher compared with the OVX group (Figure 1C). Gastrocnemius muscle weight in the OVX group was lower compared with the Sham group. Gastrocnemius muscle weights in the OVX + low‐BCAA and OVX + high‐BCAA groups were significantly higher than in the OVX group (Figure 1D).

### 3.2 BCAA Supplementation Preserves Cancellous Bone and Modulates Sclerostin, Bone Turnover Markers in OVX Mice

**FIGURE 2:**
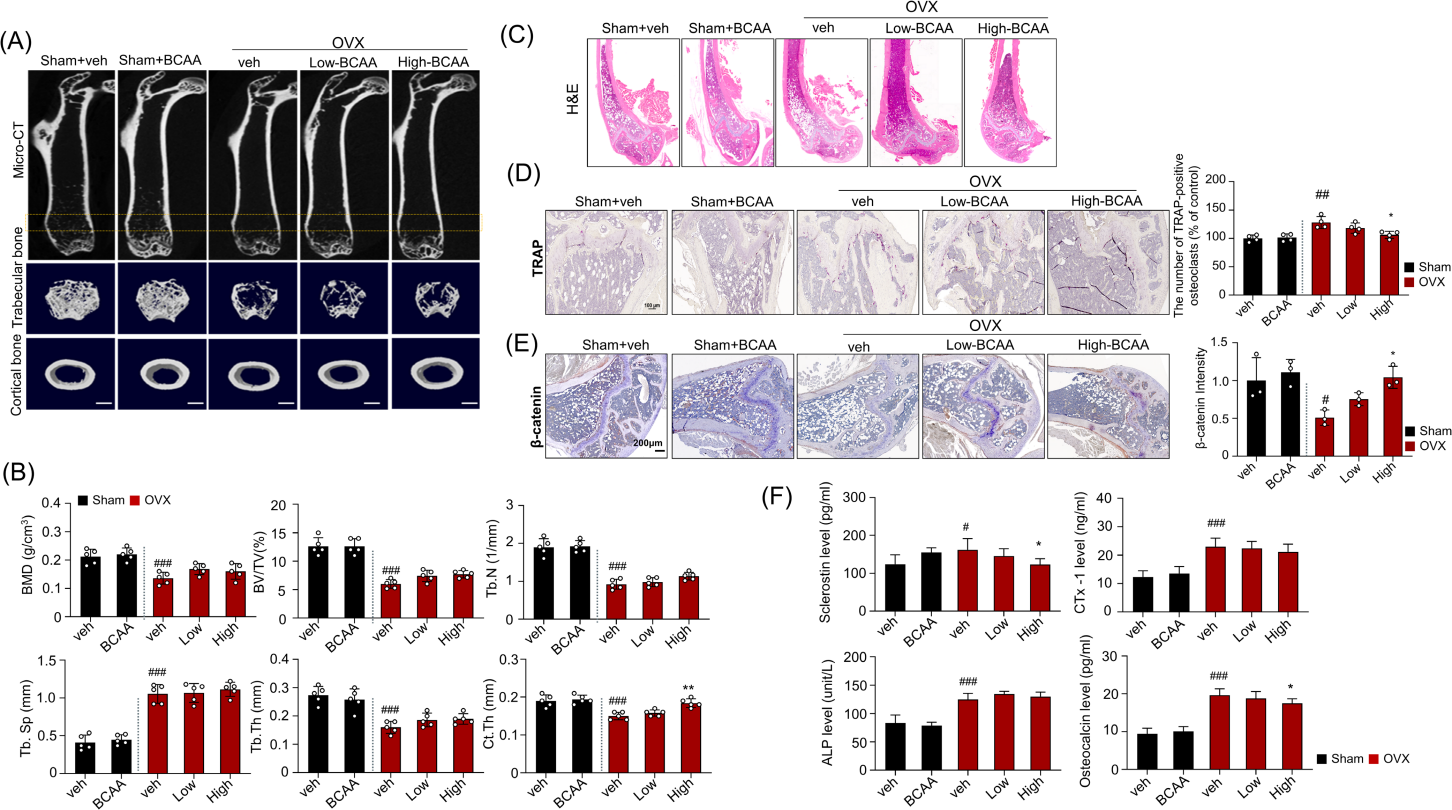
Effects of BCAA on plasma sclerostin, bone turnover markers, bone mineral density, microarchitecture and osteoclast activity in ovariectomized mice. (A) Representative micro‐CT images (top). Two‐dimensional reconstruction and three‐dimensional (3D)‐rendered images of different regions (bottom). (B) Bone structural characteristics, including bone mineral density (BMD in g/cm^3^), ratio of bone volume to total volume (BV/TV in %) and trabecular number (Tb. N in 1/mm^2^), are shown. (C) Representative H&E stained femur bone sections. (D) Representative TRAP‐stained femur bone (left) and respective quantification analysis of the TRAP‐positive osteoclasts (right). (E) Representative images and quantification of β‐catenin expression. (F) Measurement of serum levels of sclerostin, CTx‐1, ALP and osteocalcin. Data are shown as mean ± SD, *n* = 5 (B), *n* = 4 (D), *n* = 3 (E), *n* = 8 animals/group (F)). (#*p* < 0.05, ##*p* < 0.01, ###*p* < 0.001 vs. Sham + veh; *p < 0.05, **p < 0.01, ***p < 0.001 vs. OVX + veh). ALP, alkaline phosphatase; BCAA, *branched*‐chain amino acid; BCAA, Sham‐operated mice administered with 1 mg/g/day BCAA; BMD, bone mineral density; CTx‐1, β‐C‐terminal telopeptide of type 1 collagen; High, OVX mice administered with 1 mg/g of body weight/day BCAA; Low, OVX mice administered with 0.25 mg/g of body weight/day BCAA; OVX, ovariectomy; OVX, OVX mice treated with vehicle; Sham, sham‐operated group; TRAP, tartrate‐resistant acid phosphatase; Veh, Sham‐operated mice.

BMD in the whole body and femur was measured with DEXA every 4 weeks. At baseline, whole‐body BMD was comparable across all groups. However, at 4, 8, 12 and 16 weeks, the OVX group showed significantly lower whole‐body BMD compared with the Sham group (Figure S1B). However, no differences were found among OVX, OVX + low‐BCAA and OVX + high‐BCAA groups. Figure 2A shows representative micro‐CT images of the femurs from each group. Compared with the Sham group, the OVX group exhibited a marked reduction in cancellous bone mass in the distal femur metaphysis, indicating significant trabecular bone loss. Interestingly, among the OVX groups, the OVX‐BCAA group retained more trabecular bone than the untreated OVX group, suggesting a protective effect of BCAA supplementation. The 3D and cross‐sectional views clearly show preserved trabecular network and denser cortical structure in the OVX‐BCAA group. Quantitative analysis confirmed that OVX‐induced bone loss was associated with reduced BMD and deterioration of microarchitecture, whereas high‐dose BCAA treatment partially preserved bone quality, including parameters such as trabecular number (Tb.N), bone volume fraction (BV/TV) and cortical thickness (Ct.Th) (Figure 2B).

H&E staining revealed trabecular bone loss in OVX mice, which appeared to improve with high‐dose BCAA supplementation (Figure 2C). TRAP staining showed a significant increase in TRAP‐positive osteoclast numbers in the OVX group, indicating elevated bone resorption. At the same time, both low and high BCAA administration reduced the number of TRAP‐positive osteoclasts of the distal femur metaphysis activity (Figure 2D). β‐catenin expression, markedly suppressed in OVX mice, was restored with BCAA supplementation, particularly at high doses, suggesting enhanced Wnt signalling and bone remodelling potential (Figure 2E). Additionally, plasma levels of bone turnover markers were elevated in OVX mice, with significantly higher concentrations of sclerostin, CTx‐1, ALP, and osteocalcin compared with the Sham group, indicating increased bone remodelling activity. Notably, high‐dose BCAA supplementation in OVX mice reduced sclerostin and osteocalcin levels, suggesting attenuation of hyper‐sclerostinemia and a potential shift toward bone formation via enhanced Wnt/β‐catenin signalling and modulation of bone turnover dynamics (Figure 2F).

### 3.3 BCAA Alleviates OVX‐Induced Dyslipidaemia Without Affecting Liver Enzymes

OVX mice exhibited elevated fasting glucose, triglycerides and total cholesterol levels relative to Sham groups, indicating metabolic disturbances due to overiectomy. However, BCAA supplementation, particularly at high doses, significantly reduced these parameters, suggesting its potential in ameliorating OVX‐induced dyslipidaemia and hyperglycaemia. These findings support BCAA's role in improving metabolic profiles of OVX mice (Figure S2A–C). In this study, OVX in female mice led to partial worsening of metabolic dysfunctions typically associated with metabolic syndrome, such as elevated blood glucose and lipid levels. However, the liver function markers, including serum GOT and GPT levels, did not show significant differences among the various treatment groups. These observations suggest that OVX affected metabolic parameters but did not induce notable liver toxicity (Figure S2D,E).

### 3.4 Functional and Histological Improvement of Muscle With BCAA Supplementation

**FIGURE 3:**
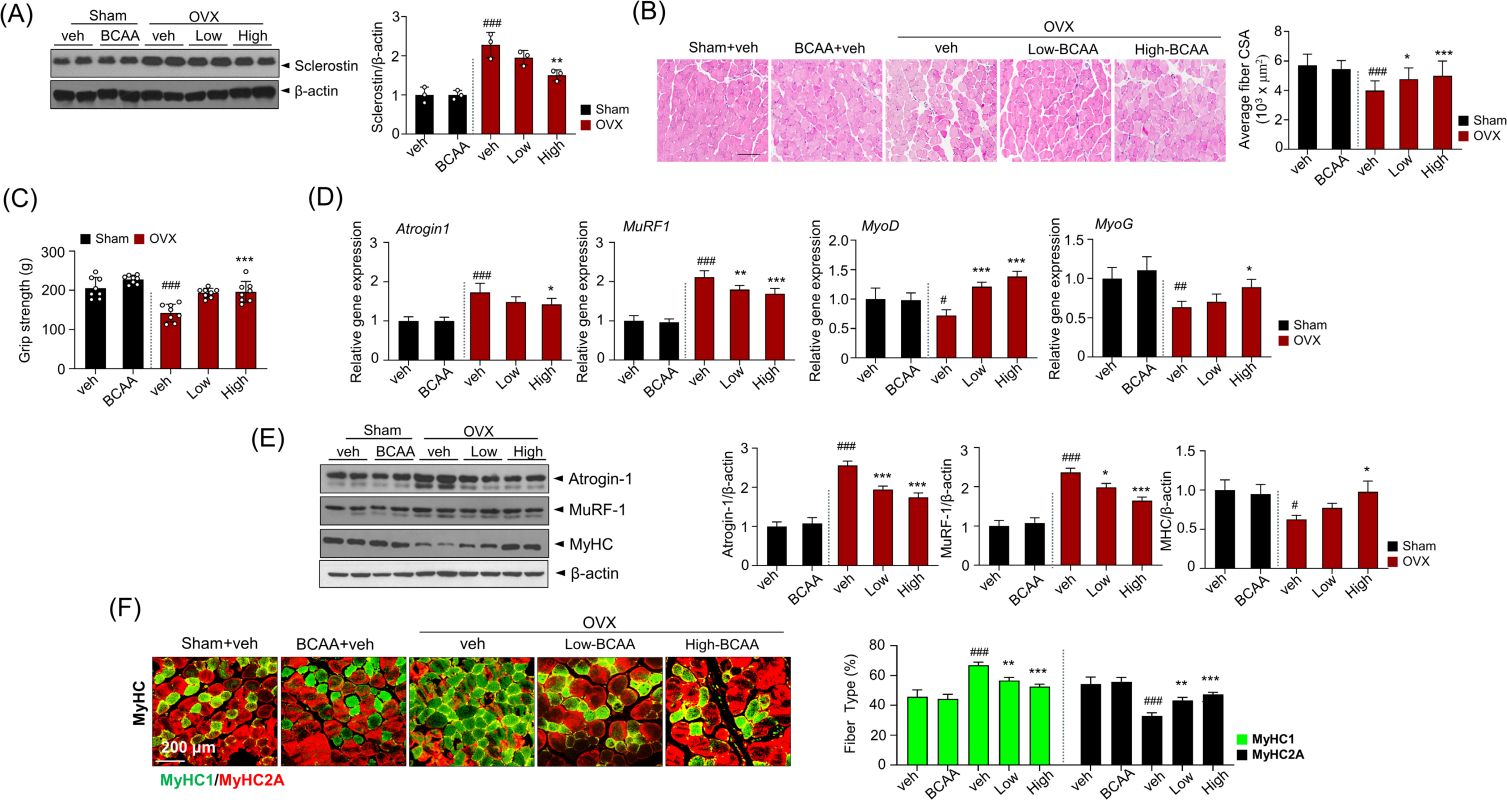
BCAA improves muscle function in ovariectomized mice. (A) Immunoblotting of Sclerostin‐1 in GAS muscle and respective quantification analysis. (B) Representative H&E staining images of the GAS muscle (left) and CSA of GAS muscle fibres (right). (C) Grip strength test. (D) qRT‐PCR analysis of muscle atrophy‐related genes (*Atrogin‐1, MuRF‐1*) and myogenesis genes (*MyoD, MyoG*) expression levels. (E) Immunoblotting using antibodies against Atrogin‐1, MuRF‐1, MHC and β‐actin in GAS muscle and their respective quantification analysis. (F) Representative image of MHC type I and II fibres immunofluorescence in GAS muscle (left) and quantitative analysis of fibre type composition (right). Data are shown as mean ± SD (*n* = 3 (A), *n* = 4 (B), *n* = 8(C), *n* = 5(D), *n* = 3 (E), *n* = 3 (F), animals/group). (#*p* < 0.05, ##*p* < 0.01, ###*p* < 0.001 vs. Sham+veh; *p < 0.05, **p < 0.01, ***p < 0.001 vs. OVX + veh). BCAA, *branched*‐chain amino acid; BCAA, Sham‐operated mice administered with 1 mg/g/day BCAA; GAS, gastrocnemius; High, OVX mice administered with 1 mg/g of body weight/day BCAA; Low, OVX mice administered with 0.25 mg/g of body weight/day BCAA; OVX, ovariectomy; OVX, OVX mice treated with vehicle; Sham, sham‐operated group; Veh, Sham‐operated mice.

In this investigation, muscle mass and strength were evaluated to show functional improvement of BCAA intake in OVX‐induced sarcopenia. To determine the effect of BCAA supplementation on sclerostin expression in skeletal muscle, sclerostin protein levels in gastrocnemius muscle tissues were assessed. The sclerostin expression was higher in OVX group. Notably, both low‐ and high‐dose BCAA supplementation markedly reduced sclerostin levels (Figure 3A). H&E staining of the gastrocnemius muscle in the Sham group revealed densely packed, well‐organised muscle fibres with numerous normal nuclei and no apparent signs of steatosis. In contrast, the gastrocnemius muscle fibres in the OVX group were disorganised, exhibiting both connective tissue proliferation and muscle steatosis, which were alleviated by BCAA administration. The CSA of the gastrocnemius muscle fibres was lower in the OVX group than in the Sham group but significantly increased following BCAA administration (Figure 3B). Further, four‐limb grip strength was improved in the BCAA group compared with the OVX group (Figure 3C).

### 3.5 BCAA Reduces Muscle Atrophy Markers and Enhances Myogenesis in OVX Mice

The relative mRNA expressions of muscle atrophy‐related genes (*Atrogin‐1* and *MuRF‐1*) and myogenesis genes (*Myo* and *MyoG*) were measured using RT‐PCR to elucidate the muscle‐preserving mechanism of BCAA oral intake. The expressions of *Atrogin‐1* and *MuRF‐1* genes were significantly higher in the OVX group compared with the Sham group. On the contrary, the expressions of Atrogin‐1 and MuRF‐1 were lower in the high‐BCAA‐intake group compared with the OVX group without BCAA (Figure 3D). In addition, *MyoD* and *MyoG* expressions were significantly lower in the OVX group than in the Sham group, while expressions were improved in the high‐BCAA‐treated groups. Besides, administration of BCAA in OVX mice regulated Atrogin‐1 and MuRF‐1 protein expressions and increased MHC protein expression (Figure 3E). Additionally, immunofluorescence was performed to assess the impact of BCAA administration on the distribution of MHC I/II of gastrocnemius, which indicated Type I, Type IIa and Type IIb fibres. In the OVX group, the density of Type IIa fibres was significantly reduced, while the density of Type I fibres was increased (Figure 3F). BCAA supplementation led to a dose‐dependent increase in the density of Type IIa fibres, which was statistically significant. Additionally, BCAA supplementation significantly increased the density of type IIb fibres compared with the OVX group. Compared with the OVX group, the pathological features of gastrocnemius in BCAA‐treated OVX mice showed significant improvements, and myocytes were orderly arranged, suggesting an anti‐sarcopenic effect of BCAA.

### 3.6 BCAA Mitigates Oxidative Stress and 4‐HNE Levels in OVX Mice

**FIGURE 4:**
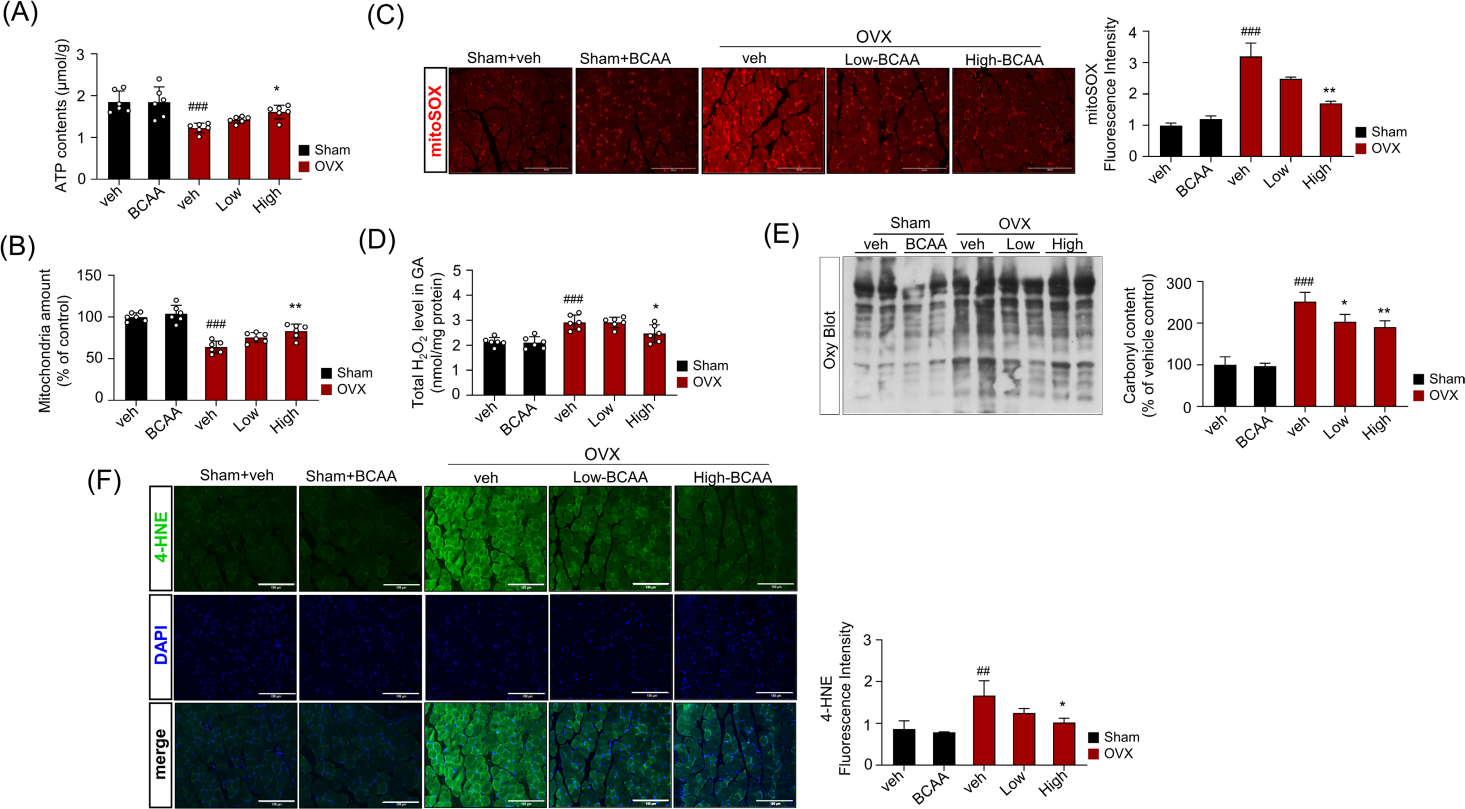
Effects of BCAA on mitochondrial function and oxidative stress in the gastrocnemius muscle of ovariectomized mice. (A) ATP levels and (B) mtDNA levels in mitochondria. (C) Representative mitoSOX fluorescence images indicating mitochondrial ROS levels in GAS muscle (left) and quantitative analysis of mitoSOX fluorescence intensity (right). (D) H_2_O_2_ levels in mitochondria and quantification of the average Amplex Red fluorescence intensity. (E) Oxy blot assay (left) and respective quantification analysis (right). (F) Representative 4HNE immunofluorescence images in GAS muscle (left) and quantitative analysis of 4HNE fluorescence intensity (right). Data are shown as mean ± SD (*n* = 6 (A, B), *n* = 3 (C), *n* = 6 (D), *n* = 3 (E, F) animals/group). (#*p* < 0.05, ##*p* < 0.01, ###*p* < 0.001 vs. Sham + veh; *p < 0.05, **p < 0.01, ***p < 0.001 vs. OVX + veh). 4HNE, 4‐hydroxynonenal; BCAA, *a branched*‐chain amino acid; BCAA, Sham‐operated mice administered with 1 mg/g/day BCAA; GAS, gastrocnemius; OVX, ovariectomy; OVX, OVX mice treated with vehicle; High, OVX mice administered with 1 mg/g of body weight/day BCAA; Low, OVX mice administered with 0.25 mg/g of body weight/day BCAA; Sham, sham‐operated group; Veh, Sham‐operated mice.

Previous investigations have shown that the abundance of mtDNA, mRNA and mitochondrial ATP production decreases during the impairment and decline of muscle functions [18]. The high‐energy skeletal muscle comprises many mitochondria, and maintaining mitochondrial integrity and function is critical for muscle health. To elucidate the mechanisms by which BCAA evokes its anti‐sarcopenic effect, mitochondrial ATP and mtDNA copy numbers per genomic DNA were evaluated in the gastrocnemius muscle of the BCAA‐administered OVX mice. The expression of mitochondrial ATP and mtDNA was significantly lower in the OVX group than in the Sham group (Figure 4A, B). However, high‐BCAA supplementation significantly restored both ATP production and mtDNA copy numbers, suggesting improved mitochondrial biogenesis and energy metabolism. Moreover, mitochondrial ROS levels assessed by mitoSOX staining were elevated in the OVX group, while the group receiving BCAA administration exhibited decreased mitochondrial ROS (Figure 4C). Additionally, OVX mice showed significantly higher levels of hydrogen peroxide (H_2_O_2_) and increased protein carbonylation, which suggests a pronounced state of oxidative stress in the skeletal muscles of OVX mice. However, BCAA supplementation significantly regulated intracellular H_2_O_2_ levels and protein carbonylation, indicating restoration of redox balance (Figure 4D–E). Further, levels of 4‐HNE, a marker of H_2_O_2_‐induced lipid peroxidation, were significantly reduced following BCAA administration (Figure 4F), indicating a protective effect against oxidative damage to membrane lipids.

### 3.7 Effects of BCAA on Muscular Cell Proliferation

**FIGURE 5:**
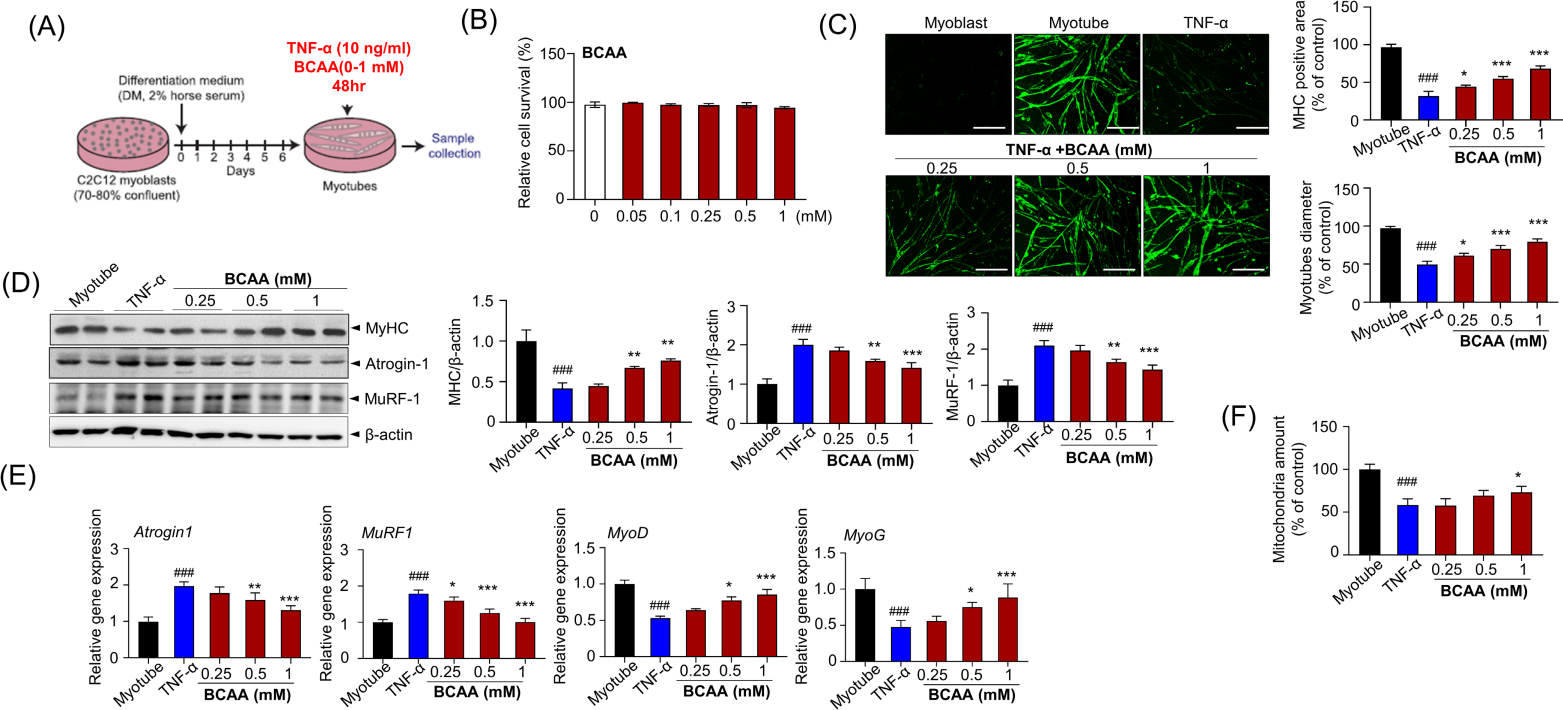
BCAA attenuates TNF‐α‐induced muscle atrophy and enhances myogenesis in C2C12 cells. (A) C2C12 cells were grown in DMEM (growth medium, GM) for 24 h and then changed to a differentiation medium (DM). Differentiated C2C12 cells were treated with BCAA (0–1 mM) and TNF‐α (10 ng/mL) for 48 h. (B) Cell viability analysis after being treated with BCAA for 24 h with different concentrations of 0, 0.05, 0.1, 0.25, 0.5 and 1 mM. (C) Representative image of MHC immunofluorescence of C2C12 myotubes. MHC positive area (top) and myotube diameter (bottom). (D) Immunoblotting using antibodies against MHC, Atrogin‐1, MuRF‐1(left) and respective quantification analysis (right). (E) qRT‐PCR analysis of muscle atrophy–related genes (*Atrogin‐1, MuRF‐1*) and myogenesis genes (*MyoD, MyoG*) expression levels. (F) mtDNA levels in mitochondria. Data are shown as mean ± SD (*n* = 3 (B,C,D), *n* = 5 (E,F), group). (^#^ *p* < 0.05, ##*p* < 0.01, ###*p* < 0.001 vs. con; *p < 0.05, **p < 0.01, ***p < 0.001 vs. TNF‐α). BCAA, a *branched*‐chain amino acid; OVX, ovariectomy.

To investigate whether BCAA alleviates muscle atrophy at the cellular level, two in vitro atrophy models were employed in C2C12 cells: TNF‐α‐induced inflammatory atrophy and dexamethasone‐induced glucocorticoid atrophy. First, in the TNF‐α model, C2C12 cells were treated with various concentrations of BCAA (0–1 mM) in the presence of 100 ng/mL TNF‐α (Figure 5A). A cell viability assay was conducted to confirm that BCAA did not induce cytotoxicity and to determine the appropriate concentration for treatment (Figure 5B). To evaluate the hypertrophic effects of BCAA, immunofluorescence staining for MHC, a structural protein of myotubes, was performed. TNF‐α treatment led to a marked reduction in MHC‐positive area and myotube diameter, indicating muscle atrophy (Figure 5C). However, BCAA supplementation restored MHC expression and increased myotube diameter in a dose‐dependent manner compared with TNF‐α‐only treated cells. In particular, high‐dose BCAA‐treated myotubes displayed stronger MHC staining and the presence of multinucleated fibres, indicating enhanced differentiation. Subsequently, the molecular mechanisms underlying these effects were investigated. Protein expression analyses showed that TNF‐α increased the expression of muscle atrophy markers, Atrogin‐1 and MuRF‐1 while decreasing MHC protein levels (Figure 5D). In contrast, BCAA treatment dose‐dependently decreased Atrogin‐1 and MuRF‐1 and increased MHC expression.

Additionally, TNF‐α suppressed the expression of myogenic regulatory genes, including *Atrogin‐1*, *MuRF‐1*, *MyoD* and *MyoG*, whereas BCAA treatment restored their levels in a dose‐dependent manner (Figure 5E). In addition, mitochondrial content, an indicator of metabolic and myogenic activity, was increased in high‐dose BCAA‐treated cells compared with TNF‐α controls (Figure 5F). Furthermore, to confirm the generalisability of BCAA's anti‐atrophic effects, a parallel experiment was performed using the dexamethasone‐induced atrophy model. C2C12 cells were treated with dexamethasone along with various concentrations of BCAA (0–1 mM) (Figure S3A). Similar to the TNF‐α model, dexamethasone treatment resulted in reduced MHC‐positive area and myotube diameter, reflecting atrophic changes (Figure S3B). Co‐treatment with BCAA significantly restored MHC expression and myotube diameter in a dose‐dependent manner. High‐dose BCAA also promoted the formation of thick, multinucleated myotubes, indicating improved myogenic fusion and differentiation. Besides, dexamethasone increased Atrogin‐1 and MuRF‐1 protein expression and decreased MHC levels, while BCAA treatment reversed these effects in a dose‐dependent fashion (Figure S3C). Collectively, these results demonstrate that BCAA supplementation exerts protective effects against both inflammatory and glucocorticoid‐induced muscle atrophy by suppressing atrophy‐related gene expression and enhancing myogenic differentiation and mitochondrial activity.

### 3.8 BCAA Positively Regulates the Sclerostin Secretion in MLO‐Y4 and C2C12 Cells

**FIGURE 6:**
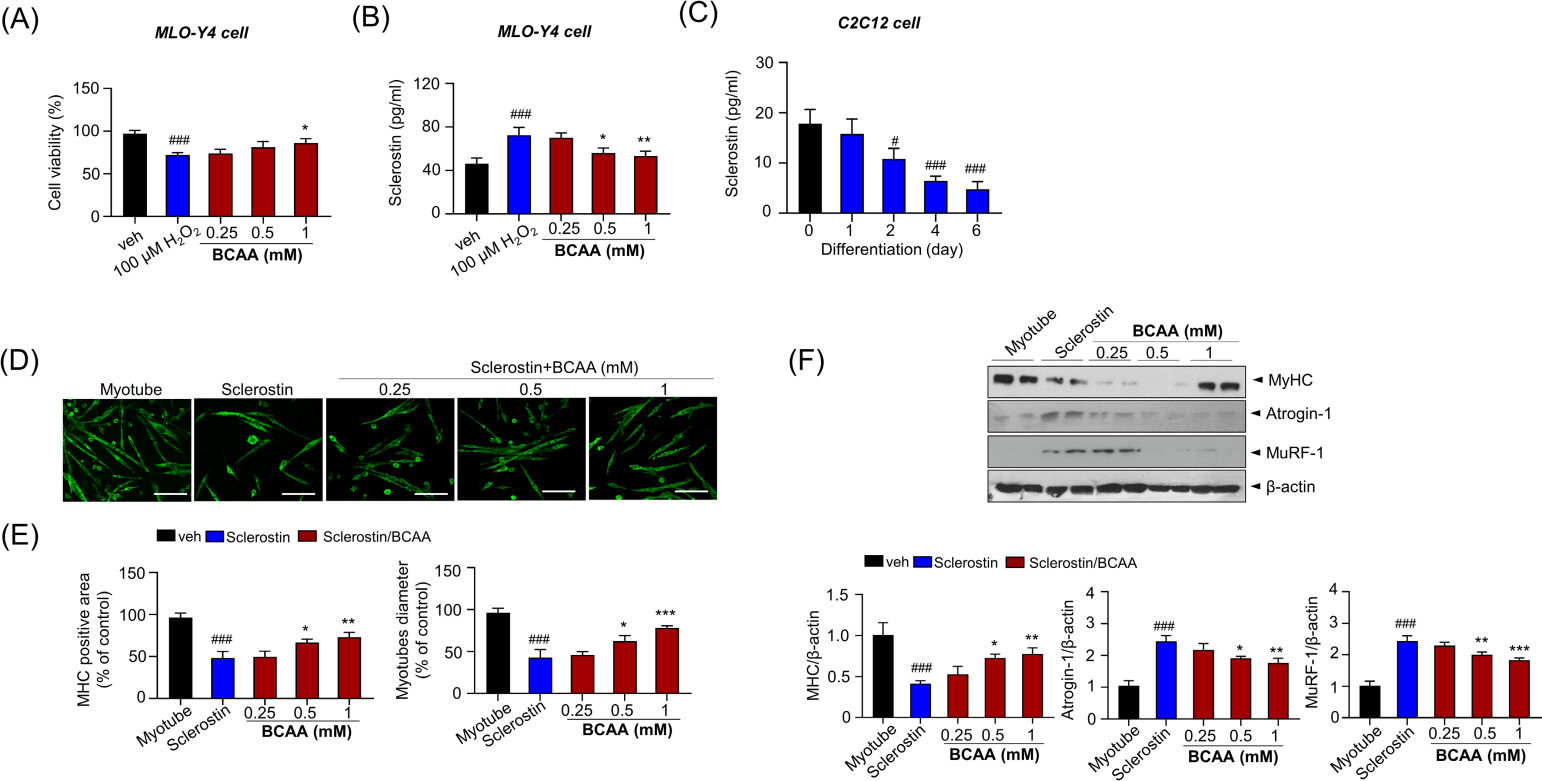
BCAA modulates sclerostin levels in MLO‐Y4 and C2C12 cells under oxidative stress and during myogenic differentiation. MLO‐Y4 cells were treated with 100 μM H_2_O_2_ and BCAA (0.25, 0.5 and 1 mM) for 6 h. Cell viability (A) and the level of sclerostin (B) were measured in the MLO‐Y4 cells. C2C12 cells were under the differentiation condition and the level of sclerostin was measured in the C2C12 cells. Differentiated C2C12 cells were treated with 100 ng/mL sclerostin under the indicated concentrations of BCAA during the last 2 days. Representative image of MHC immunofluorescence of C2C12 myotubes (D). MHC positive area and myotube diameter (E). (F) Immunoblotting using antibodies against MHC, Atrogin‐1 and MuRF‐1 and respective quantification analysis (bottom). Data are shown as mean ± SD (*n* = 3, #*p* < 0.05, ##*p* < 0.01, ###*p* < 0.001 vs. Myotube; *p < 0.05, **p < 0.01, ***p < 0.001 vs. Sclerostin).

The protective effects of BCAA against H_2_O_2_‐induced cytotoxicity were evaluated in primary osteocytes, MLO‐Y4 cells. BCAA improved the viability of MLO‐Y4 cells, which represent impaired osteocytic function by H_2_O_2_ treatment (Figure 6A). RNA analysis in MLO‐Y4 osteocytic cells exposed to H_2_O_2_‐induced oxidative stress revealed a significant increase in *Sost* (the gene encoding sclerostin) expression, while Wnt signalling–related genes (*Axin2*, *Lef1* and *Ctnnb*) showed a trend of compensatory upregulation, possibly reflecting a feedback response (Figure S4). Upon H_2_O_2_ exposure, sclerostin protein levels were markedly increased, whereas BCAA dose‐dependently attenuated this elevation (Figure 6B). To explore the role of sclerostin in muscle physiology, we monitored its expression during myogenic differentiation in C2C12 cells. Sclerostin expression progressively decreased as differentiation advanced (Figure 6C), implying an inverse relationship with myogenesis.

Furthermore, exogenous sclerostin treatment significantly reduced the MyHC‐positive area and myotube diameter, indicating impaired myogenic differentiation (Figure 6D, E). Notably, co‐treatment with BCAA dose‐dependently reversed these suppressive effects of sclerostin. Western blot analysis confirmed that BCAA restored MyHC expression while reducing Atrogin‐1 and MuRF1 levels in sclerostin‐treated C2C12 cells (Figure 6F), supporting a role for BCAA in counteracting sclerostin‐induced muscle atrophy and promoting myotube formation.

## 4 Discussion

This study investigated the effects of BCAA supplementation on body composition, bone mineral density (BMD), muscle function and mitochondrial health in OVX mice, with a focus on the modulation of sclerostin, a key negative regulator of bone metabolism. Our results suggest that BCAA supplementation protects against muscle atrophy, improves mitochondrial function and modulates sclerostin levels. However, its overall impact on bone mineral density was limited.

### 4.1 Sclerostin and Its Role in Bone Metabolism

Sclerostin, primarily secreted by osteocytes, inhibits the Wnt/β‐catenin signalling pathway and negatively regulates bone formation. Consistent with previous reports [19, 20, 21], serum sclerostin levels were significantly elevated in OVX mice (Figure 2B), which is characteristic of postmenopausal osteoporosis. BCAA supplementation significantly reduced circulating sclerostin levels, potentially alleviating its inhibition on Wnt signalling and partially restoring osteoblast activity and bone microarchitecture.

Immunohistochemical analysis further confirmed that β‐catenin expression increased in a dose‐dependent manner in the trabecular bone region of BCAA‐treated OVX mice (Figure 2E). To further clarify this mechanism, we examined mRNA expression in H_2_O_2_‐treated MLO‐Y4 osteocytes, mimicking oestrogen deficiency–related oxidative stress. H_2_O_2_ increased *Sost* expression and downregulated Wnt target genes (*Axin2*, *Lef1* and *Ctnnb1*). BCAA reversed these changes, restoring Wnt pathway activity (Figure S4). These results support the idea that oxidative stress elevates sclerostin, and BCAA attenuates this response, thereby preserving osteocytic Wnt signalling.

### 4.2 Muscle and Bone Cross‐Talk Through Sclerostin

Mechanical loading is well‐established physiological stimulus that play crucial role in maintaining bone homeostasis. It is extensively reported that mechanical forces suppress sclerostin expression, enhancing Wnt‐mediated bone formation [22]. In this study, BCAA supplementation exerted a comparable effect by improving muscle function and reducing sclerostin levels, which may indirectly influence bone metabolism. Notably, this reduction in sclerostin was accompanied by muscle hypertrophy and functional improvement, as shown by increased CSA of gastrocnemius fibres and enhanced grip strength (Figure 3D, Figure 3E). These observations align with previous studies showing that biochemical signals from muscle activity can downregulate sclerostin and stimulate bone formation [23]. Thus, enhanced muscle function following BCAA supplementation could contribute to reduced sclerostin expression, supporting bone homeostasis even in the absence of significant BMD improvement.

Previous studies show that sclerostin antagonises canonical Wnt signalling via LRP5/6 and suppresses Wnt3a‐driven myogenic differentiation in C2C12 cells and exogenous sclerostin significantly reduced Wnt3a‐induced myotube formation/MyHC staining in C2C12, while Wnt3a increased β‐catenin nuclear translocation during myogenesis [24, 25]. These data align with mechanistic work demonstrating direct inhibition of Wnt‐induced β‐catenin signalling by sclerostin and with genetic/pharmacologic evidence that neutralising sclerostin augments Wnt activity in vivo [26, 27, 28]. Together with the central role of Wnt signalling in skeletal myogenesis, these studies support our interpretation that down‐modulation of sclerostin relieves Wnt pathway inhibition to facilitate myogenic progression.

To further investigate this mechanism, we assessed sclerostin expression under oxidative stress in MLO‐Y4 osteocytes. H_2_O_2_ exposure increased sclerostin levels, which were dose‐dependently reduced by BCAA treatment (Figure 6B). Additionally, during C2C12 myoblast differentiation, sclerostin expression progressively declined (Figure 6C), suggesting a physiological suppression of sclerostin during muscle development. Exogenous sclerostin impaired myogenic differentiation, whereas BCAA reversed these effects (Figure 6D–F), indicating that BCAA‐mediated sclerostin suppression supports both osteocytic and myogenic health.

Collectively, these findings highlight a dual regulatory role of BCAAs in muscle and bone metabolism via sclerostin modulation. Further research is needed to elucidate the specific molecular pathways involved in this muscle–bone crosstalk.

### 4.3 Limited Impact on Bone Mineral Density

Oestrogen deficiency following menopause elevates sclerostin expression, partly via increased ROS production in osteocytes, which upregulates the SOST gene. In this study, oxidative stress induced sclerostin secretion in MLO‐Y4 osteocytes, while BCAA treatment improved cell viability and reduced sclerostin levels (Figure 6A,B). Despite these effects, BCAA supplementation did not significantly improve BMD in OVX mice (Figure 1C; Figure S1). These findings suggest that although BCAA modulates sclerostin and may influence bone metabolism, its overall impact on BMD is limited—likely due to persistent osteoclast activation and bone resorption under oestrogen‐deficient conditions [29]. Prior studies also show that sclerostin suppression alone cannot fully restore bone density without concurrently inhibiting osteoclastic activity [30]. Anti‐sclerostin antibodies such as romosozumab are known to partially alleviate osteoclastic bone resorption by weakly acting on the RANKL pathway [29]. Therefore, the findings of this study indicate that BCAA supplementation potentially reduce metaphyseal bone resorption by suppressing osteoclastic activity through decreasing sclerostin secretion. However, the anti‐sclerostin activity of BCAA alone could be insufficient to induce osteoblastic bone formation through the Wnt/β‐catenin pathway. While anti‐sclerostin agents like romosozumab weakly reduce resorption via the RANKL pathway, BCAA may offer similar partial benefits by decreasing sclerostin secretion. However, this may be insufficient to stimulate osteoblastic bone formation through Wnt/β‐catenin signalling. The femoral and whole‐body BMD in OVX mice remained low, underscoring the limited efficacy of BCAA alone in preventing bone loss. Combination strategies with antiresorptive (e.g., bisphosphonates, denosumab) or anabolic therapies (e.g., teriparatide, romosozumab) may yield greater skeletal benefits [31]. Further studies could explore these combinations to optimise both muscle and bone health.

### 4.4 Plasma Enzyme Levels and Bone Turnover

The plasma levels of bone turnover markers, such as CTx‐1 and osteocalcin, were elevated in OVX mice compared with the sham group, indicating increased bone turnover. Notably, BCAA supplementation lowered these markers (Figure 4C,D), suggesting that BCAAs may modulate bone remodelling processes. However, these biochemical changes did not translate into significant changes in BMD (Figure 1C; Figure S1). This result emphasised the complex interplay between bone turnover and bone mineral density, where the extent of reducing serum sclerostin level alone may be inadequate to inhibit bone loss, especially in oestrogen‐deficient conditions.

### 4.5 Mitochondrial Health and Muscle Function

In this investigation, BCAA supplementation improved mitochondrial function, as evidenced by increased ATP production, mitochondrial DNA content and reduced oxidative stress (Figure 5A–C). These findings are consistent with the overall improvements in muscle function, including increased grip strength and muscle fibre size (Figure 3D,E). Together, observations of the study support the role of BCAAs in enhancing muscle health, potentially through the modulation of sclerostin and Wnt signalling, further highlighting the interconnectivity between muscle and bone.

### 4.6 Implication for Therapeutic Strategies

Given the role of sclerostin in bone metabolism, study findings suggest that BCAA supplementation could serve as an adjunct therapy for conditions like osteoporosis, where elevated sclerostin levels contribute to bone loss [32]. By reducing sclerostin levels, BCAA supplementation may enhance the efficacy of other therapeutic interventions, particularly those that stimulate bone formation or inhibit bone resorption [31]. Additionally, the observed improvement in muscle function through reduced sclerostin levels highlights the potential for BCAAs to be part of a broader strategy for managing osteosarcopenia. Such management strategies are consistent with the method of targeting both muscle and bone health simultaneously, primarily through pathways like Wnt signalling, which may offer more effective treatments for aging populations at risk of sarcopenia and osteoporosis [32]. Although gain‐ or loss‐of‐function studies were not performed in this study, existing evidence suggests that sclerostin may suppress Wnt‐mediated myogenesis. Future investigation is warranted to elucidate whether the downregulation of sclerostin is necessary for myoblast differentiation and whether it directly influences myoblast proliferation or survival.

### 4.7 Limitations and Conclusion

Although anti‐sclerostin antibodies are clinically used for treating severe osteoporosis, this study did not confirm a direct anti‐sclerostin effect of BCAA intake in the human bone–muscle system. The dose‐ and time‐dependent efficacy of BCAA remains unclear, as does its ability to improve osteopenia and sarcopenia either independently or synergistically. Optimising BCAA administration may require careful adjustment of timing, concentration and duration, especially in oestrogen‐deficient or aging populations. BCAA supplementation did not affect liver function and showed metabolic benefits by improving dyslipidaemia and glucose levels. These findings suggest potential systemic effects, but the impact on other organs requires further investigation.

In conclusion, this study highlights sclerostin as a key mediator in muscle and bone metabolism. BCAA supplementation reduced serum sclerostin levels and supported Wnt signalling under oestrogen‐deficient conditions, leading to improved muscle health. However, its effect on BMD was limited. Future studies should explore long‐term efficacy and evaluate combination strategies with anabolic or anti‐resorptive agents. Molecular insights into sclerostin‐mediated muscle–bone crosstalk may further aid in developing novel therapies for osteosarcopenia.

## Conflicts of Interest

The authors declare no conflicts of interest.

## Supporting information


**Table S1:** qPCR primers used in the study.
Figure. S1. In vivo assessment of BMD changes and time‐dependent changes in lean mass index following BCAA treatment. The changes in lean mass index (lean mass/body weight) from baseline to the end of the intervention as detected by DEXA at 4, 8, 12 and 16 weeks after BCAA supplementation (A). The changes in BMD from baseline to the end of the intervention, as detected by DEXA at 4, 8, 12 and 16 weeks after BCAA supplementation (B). Data are shown as mean ± SD (*n* = 10 (A), *n* = 8 (B)/animals/group). (#*p* < 0.05, ##*p* < 0.01, ###*p* < 0.001 vs. Sham + veh; *p < 0.05, **p < 0.01, ***p < 0.001 vs. OVX + veh). Sham, sham‐operated group; OVX, ovariectomy; BCAA, *branched*‐chain amino acid. Veh, Sham‐operated mice; BCAA, Sham‐operated mice administered with 1 mg/g/day BCAA; OVX, OVX mice treated with vehicle; Low,  OVX mice administered with 0.25 mg/g of body weight/day BCAA; High, OVX mice administered with 1 mg/g of body weight/day BCAA.
Figure. S2 Influence of BCAA on glycolipid metabolism and GOT and GPT serum levels in ovariectomised mice. A biochemical analyser was used to determine the blood levels of (A) fasting glucose level, (B) triglycerides and (C) total cholesterol. (D, E) Serum levels of GOT and GPT in ovariectomised mice with or without BCAA supplementation to evaluate liver function. Data are shown as mean ± SD (*n* = 8 animals/group). (#*p* < 0.05, ##*p* < 0.01, ###*p* < 0.001 vs. Sham + veh; *p < 0.05, **p < 0.01, ***p < 0.001 vs. OVX + veh). BCAA, *branched*‐chain amino acid; BCAA, Sham‐operated mice administered with 1 mg/g/day BCAA; GOT, glutamate oxaloacetate transaminase; GPT, glutamate pyruvate transaminase; High, OVX mice administered with 1 mg/g of body weight/day BCAA; Low, OVX mice administered with 0.25 mg/g of body weight/day BCAA; OVX, ovariectomy; OVX, OVX mice treated with vehicle; Sham, sham‐operated group; Veh, Sham‐operated mice.
Figure. S3 BCAA attenuates dexamethasone‐induced muscle atrophy and enhances myogenesis in C2C12 Cells. (A) C2C12 myoblasts were cultured to 70%–80% confluence, differentiated in DM for 6 days, and then treated with dexamethasone (10 μM) and BCAA (0–1 mM) for 24 h. (B) Myosin heavy chain (MHC) immunofluorescence staining was performed to evaluate myotube morphology. Quantification of MHC‐positive area and myotube diameter is shown. (C) Western blotting was conducted to assess MHC, Atrogin‐1 and MuRF‐1 protein levels, with densitometric analysis. Data are shown as mean ± SD (*n* = 5 (B), *n* = 3 (C), group). (#*p* < 0.05, ##*p* < 0.01, ###*p* < 0.001 vs. Myotube; *p < 0.05, **p < 0.01, ***p < 0.001 vs. dexamethasone).
Figure. S4 BCAA modulates sclerostin and Wnt pathway gene expression under oxidative stress. mRNA levels of *Sost*, *Axin2*, *Lef1* and *Catnb* measured in MLO‐Y4 osteocytic cells after H_2_O_2_ exposure with or without BCAA treatment using qRT‐PCR. Data are shown as mean ± SD (*n* = 3, #*p* < 0.05, ##*p* < 0.01, ###*p* < 0.001 vs. Myotube; *p < 0.05, **p < 0.01, ***p < 0.001 vs. H_2_O_2_).
