# Supplementary material for: Role of Branched‐Chain Amino Acids in Mitigating Osteosarcopenia: An Experimental Study Using Ovariectomised Mice Models
Source: J Cachexia Sarcopenia Muscle. 2025 Oct 29;16(6):e70105. doi: 10.1002/jcsm.70105 (PMC12569608; doi:10.1002/jcsm.70105)
Supplement: Supplementary file 1 — Table S1: qPCR primers used in the study. Figure. S1. In vivo assessment of BMD changes and time‐dependent changes in lean mass index following BCAA treatment. The changes in lean mass index (lean mass/body weight) from baseline to the end of the intervention as detected by DEXA at 4, 8, 12 and 16 weeks after BCAA supplementation (A). The changes in BMD from baseline to the end of the intervention, as detected by DEXA at 4, 8, 12 and 16 weeks after BCAA supplementation (B). Data are shown as mean ± SD (n = 10 (A), n = 8 (B)/animals/group). (#p < 0.05, ##p < 0.01, ###p < 0.001 vs. Sham + veh; *p < 0.05, **p < 0.01, ***p < 0.001 vs. OVX + veh). Sham, sham‐operated group; OVX, ovariectomy; BCAA, branched‐chain amino acid. Veh, Sham‐operated mice; BCAA, Sham‐operated mice administered with 1 mg/g/day BCAA; OVX, OVX mice treated with vehicle; Low, OVX mice administered with 0.25 mg/g of body weight/day BCAA; High, OVX mice administered with 1 mg/g of body weight/day BCAA. Figure. S2 Influence of BCAA on glycolipid metabolism and GOT and GPT serum levels in ovariectomised mice. A biochemical analyser was used to determine the blood levels of (A) fasting glucose level, (B) triglycerides and (C) total cholesterol. (D, E) Serum levels of GOT and GPT in ovariectomised mice with or without BCAA supplementation to evaluate liver function. Data are shown as mean ± SD (n = 8 animals/group). (#p < 0.05, ##p < 0.01, ###p < 0.001 vs. Sham + veh; *p < 0.05, **p < 0.01, ***p < 0.001 vs. OVX + veh). BCAA, branched‐chain amino acid; BCAA, Sham‐operated mice administered with 1 mg/g/day BCAA; GOT, glutamate oxaloacetate transaminase; GPT, glutamate pyruvate transaminase; High, OVX mice administered with 1 mg/g of body weight/day BCAA; Low, OVX mice administered with 0.25 mg/g of body weight/day BCAA; OVX, ovariectomy; OVX, OVX mice treated with vehicle; Sham, sham‐operated group; Veh, Sham‐operated mice. Figure. S3 BCAA attenuates dexamethasone‐induced muscle atrophy and enhances myogene [file JCSM-16-e70105-s001.docx]

Supplemental Experimental Procedures

*Quantitative RT-PCR*

Real-time polymerase chain reaction assay (RT-PCR) was performed as described previously [16]. Briefly, homogenized C2C12 cells and skeletal muscle tissue were used for RNA extraction using the TRIzol solution (Invitrogen Corporation). Isolated RNA (2 μg) was used for cDNA synthesis using oligo dT primers and reverse transcriptase. The reaction mixture was prepared according to the manufacturer's instructions (TaKaRa SYBR Premix Ex Taq Kit, TaKaRa Bio Inc., Tokyo, Japan). The reaction was performed on an ABI 7500 Real-Time PCR system (Applied Biosystems, Foster City, CA, USA). The amplified products were quantified using the comparative cycle threshold (Ct) method. Primers used in this study are listed below. Primers used in this study are listed in Table S1.

***Immunoblotting***

Immunoblotting was performed as described previously. Briefly, C2C12 cells and skeletal muscle tissue lysates were sonicated, separated on a polyacrylamide gel, and transferred onto PVDF membranes. Blocked membranes were proven with primary antibodies against Sclerostin, Atrogin-1, MuRF-1, MHC, and β-actin (Santa Cruz Biotechnologies, CA, USA, sc-518161, sc-166806, sc-27643, sc-55582, sc-47778). Blots were washed with TBS-T buffer, blocked with 5% Skim milk or BSA for 1 h, and probed again with species-specific horseradish peroxidase (HRP)-conjugated secondary antibodies. Protein signals were developed using ECL (Bio-Rad, Hercules, CA, USA), and their quantifications were performed by measuring the band intensity using the Image J analysis (NIH).

###### *Assessment of bone microstructure using micro-computed tomography*

Micro-CT scanning was performed on left femur samples using a SKYSCAN 1076 Micro-CT (SkyScan, Kontich, Belgium) installed in the Center for University-Wide Research Facilities (CURF) at Jeonbuk National University. Mice bone scanning was performed at 8 μm pixel size, using an X-ray source 75 kV, 100 mA. Raw images were reconstructed using the SkyScan reconstruction software (NRecon) to create 3-dimensional (3D) cross-sectional image datasets using a 3D cone-beam algorithm. Structural indices were computed on reconstructed images using the Skyscan CT Analyzer (CTAn) software (Bruker). Also, based on the thickness, cortical and trabecular bone were separated using a custom processing algorithm in CTAn. Each sample was analyzed for trabecular parameters, including BMD, trabecular number (Tb. N), trabecular thickness (Tb. Th), bone volume to tissue volume (BV/TV), cortical thickness, and trabecular separation (Tb. Sp).

***Measurement of mitochondrial functions***

ATP levels in the left gastrocnemius muscle were measured with a luciferin-luciferase bioluminescence assay kit for ATP synthase enzyme activity (Abcam, Cambridge, UK), following the manufacturer’s instructions. Results are reported as µmol ATP per gram of tissue (µmol g⁻¹). **Mitochondrial DNA (mtDNA) was also extracted from the gastrocnemius muscle** using the RNeasy Mini Kit (Qiagen, Hilden, Germany; Cat. No. 74104) according to the manufacturer’s protocol. Relative mtDNA content was quantified by qPCR targeting cytochrome c oxidase subunit I (Cox 1), with 18S rRNA serving as the reference gene. The primer sequences were as follows: Cox 1 forward 5′- TGCTAGCCGCAGGCATTACT -3′ and reverse 5′- CGGGATCAAAGAAAGTTGTGT -3′; 18S rRNA forward 5′- CGCGGTTCTATTTTGTTGGT -3′ and reverse 5′- AGTCGGCATCGTTTATGGTC -3′. Relative mtDNA levels were calculated using the comparative Ct (ΔΔCt) method.

***Measurement of ROS and oxidative stress assay***

The H₂O₂ levels in mitochondria and muscle tissue were quantitatively detected by an Amplex red kit (Invitrogen Corporation, A22188). Protein oxidation was measured in 5 µg of muscle extracts using the OxyBlot^TM^ Protein Oxidation Detection kit (Merck Millipore, Darmstadt, Germany; Cat. No. S7150), following the manufacturer guidelines.

***Immunoassay of plasma enzymes***

A bone resorption marker in plasma, β-C-terminal telopeptide of type 1 collagen (CTx-1, #MBS9901663), along with osteocalcin (#MBS2020904) and alkaline phosphatase (ALP, #MBS2087848), were measured using a Mouse ELISA Kit (MyBioSource, San Diego, CA, USA). The levels of sclerostin in both cell culture medium and mouse plasma were determined using the relevant Mouse Sclerostin ELISA Kit (#MSST00, R&D Systems, Minneapolis, MN, USA).

***Tartrate-resistant acid phosphatase (TRAP) assay***

For cytochemical staining of TRAP-positive cells, deparaffinized tissue sections or 10% formaldehyde-fixed cells were stained for TRAP following the manufacturer's protocol. TRAP-positive cells were counted microscopically (Olympus Optical Co.Ltd, Japan), and their activity was determined as described previously [19].

**Supplementary Table S1. qPCR primers used in the study.**

| **Genes** | **Forward (5' to 3')** | **Reverse (5' to 3')** |
| --- | --- | --- |
| *MyoG* | GAGACATCCCCCTATTTCTACCA | GCTCAGTCCGCTCATAGCC |
| *MyoD* | AGCACTACAGTGGCGACTCAG | AGGCGGTGTCGTAGCCATTC |
| *Atrogin1* | GTCGCAGCCAAGAAGAGAAAGA | TGCTATCAGCTCCAACAGCCTT |
| *MuRF1* | TAACTGCATCTCCATGCTGGTG | TGGCGTAGAGGGTGTCAAACTT |
| *Sost* | AGCCTTCAGGAATGATGCCAC | CTTTGGCGTCATAGGGATGGT |
| *Axin2* | TGACTCTCCTTCCAGATCCCA | TGCCCACACTAGGCTGACA |
| *Lef1* | TGTTTATCCCATCACGGGTGG | CATGGAAGTGTCGCCTGACAG ( |
| *Catnb* | ATGGAGCCGGACAGAAAAGC | CTTGCCACTCAGGGAAGGA |
| *GAPDH* | CATCTTCCAGGAGCGAGACC | TGAAGTCGCAGGAGACAACC |


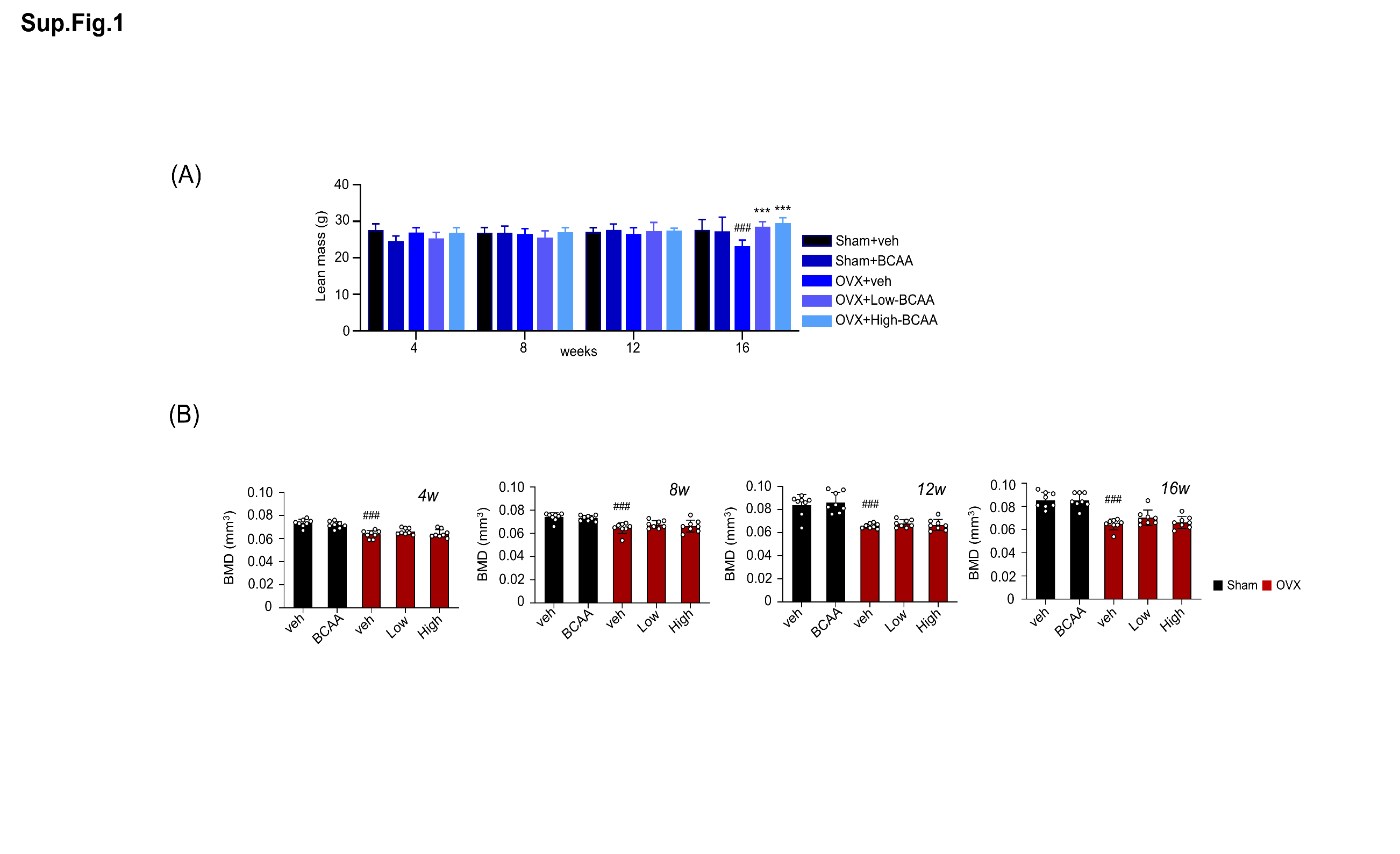
Supplementary Figure. 1. *In vivo* assessment of BMD changes and Time-dependent changes in lean mass index following BCAA treatment. The changes in lean mass index (lean mass/body weight) from baseline to the end of the intervention as detected by DEXA at 4, 8, 12, and 16 weeks after BCAA supplementation (A). The changes in BMD from baseline to the end of the intervention, as detected by DEXA at 4, 8, 12, and 16 weeks after BCAA supplementation (B). Data are shown as mean ± SD (n=10 (A), n=8 (B)/ animals/group). ( ^#^*p* < 0.05, ^##^*p* < 0.01, ^###^*p* < 0.001 vs Sham+veh ; ^*^*p* < 0.05, ^**^*p* < 0.01, ^***^*p* < 0.001 vs. OVX+veh). Sham, sham-operated group; OVX, ovariectomy; BCAA, branched-chain amino acid. Veh, Sham-operated mice; BCAA, Sham-operated mice administered with 1 mg/g/day BCAA; OVX, OVX mice treated with vehicle; Low, OVX mice administered with 0.25 mg/g of body weight/day BCAA; High, OVX mice administered with 1 mg/g of body weight/day BCAA.


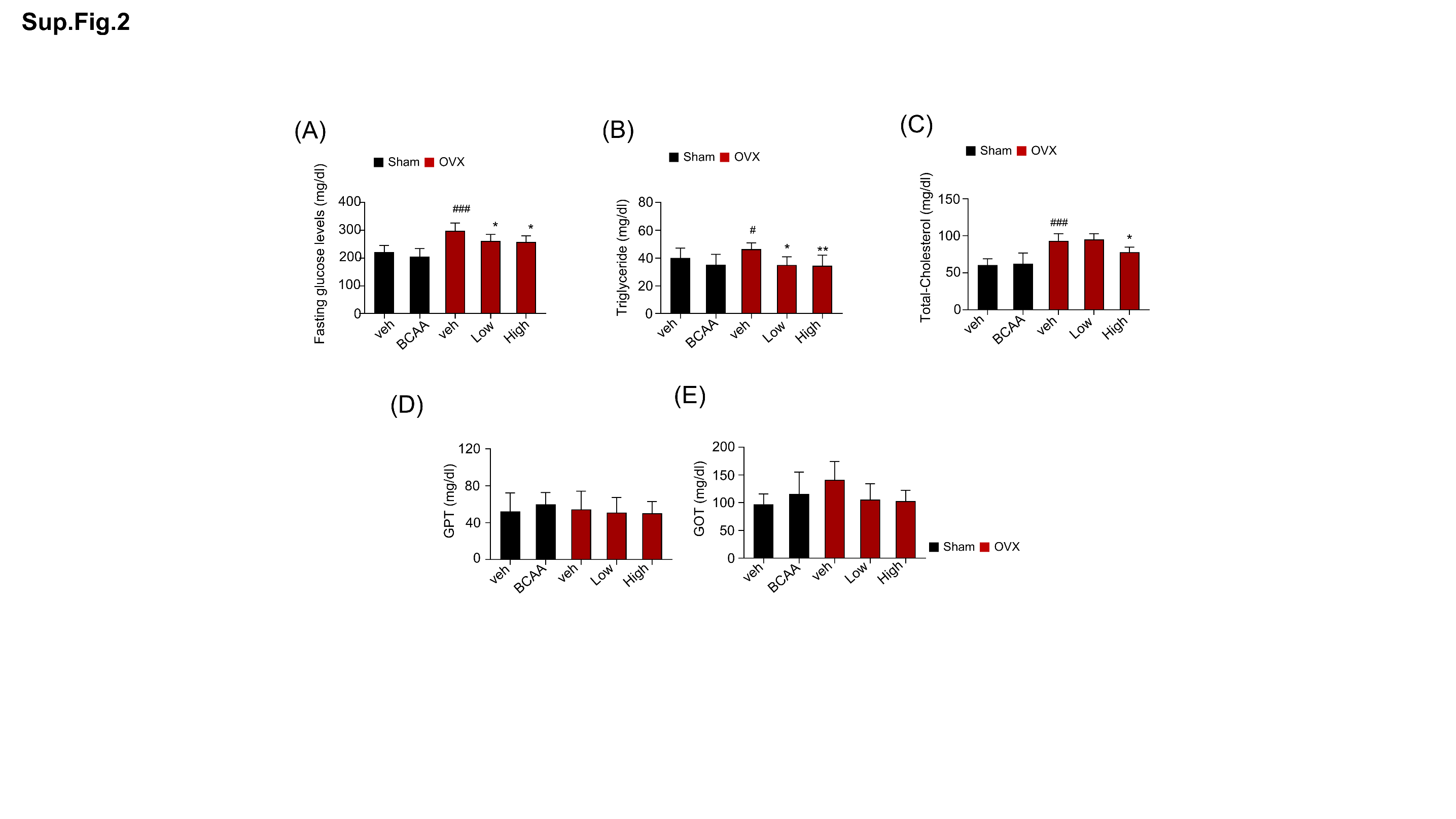


**Supplementary Figure. 2 Influence of BCAA on glycolipid metabolism and GOT and GPT serum levels in ovariectomized** **mice.** A biochemical analyzer was used to determine the blood levels of (A) Fasting glucose level, (B) triglycerides, and (C) total cholesterol. (D-E) Serum levels of GOT and GPT in ovariectomized mice with or without BCAA supplementation to evaluate liver function. Data are shown as mean ± SD (n=8 animals/group). ( ^#^*p* < 0.05, ^##^*p* < 0.01, ^###^*p* < 0.001 vs Sham+veh ; ^*^*p* < 0.05, ^**^*p* < 0.01, ^***^*p* < 0.001 vs. OVX+veh). Sham, sham-operated group; OVX, ovariectomy; BCAA, branched-chain amino acid; GOT, Glutamate Oxaloacetate Transaminase; GPT, Glutamate Pyruvate Transaminase. Veh, Sham-operated mice; BCAA, Sham-operated mice administered with 1 mg/g/day BCAA; OVX, OVX mice treated with vehicle; Low, OVX mice administered with 0.25 mg/g of body weight/day BCAA; High, OVX mice administered with 1 mg/g of body weight/day BCAA.


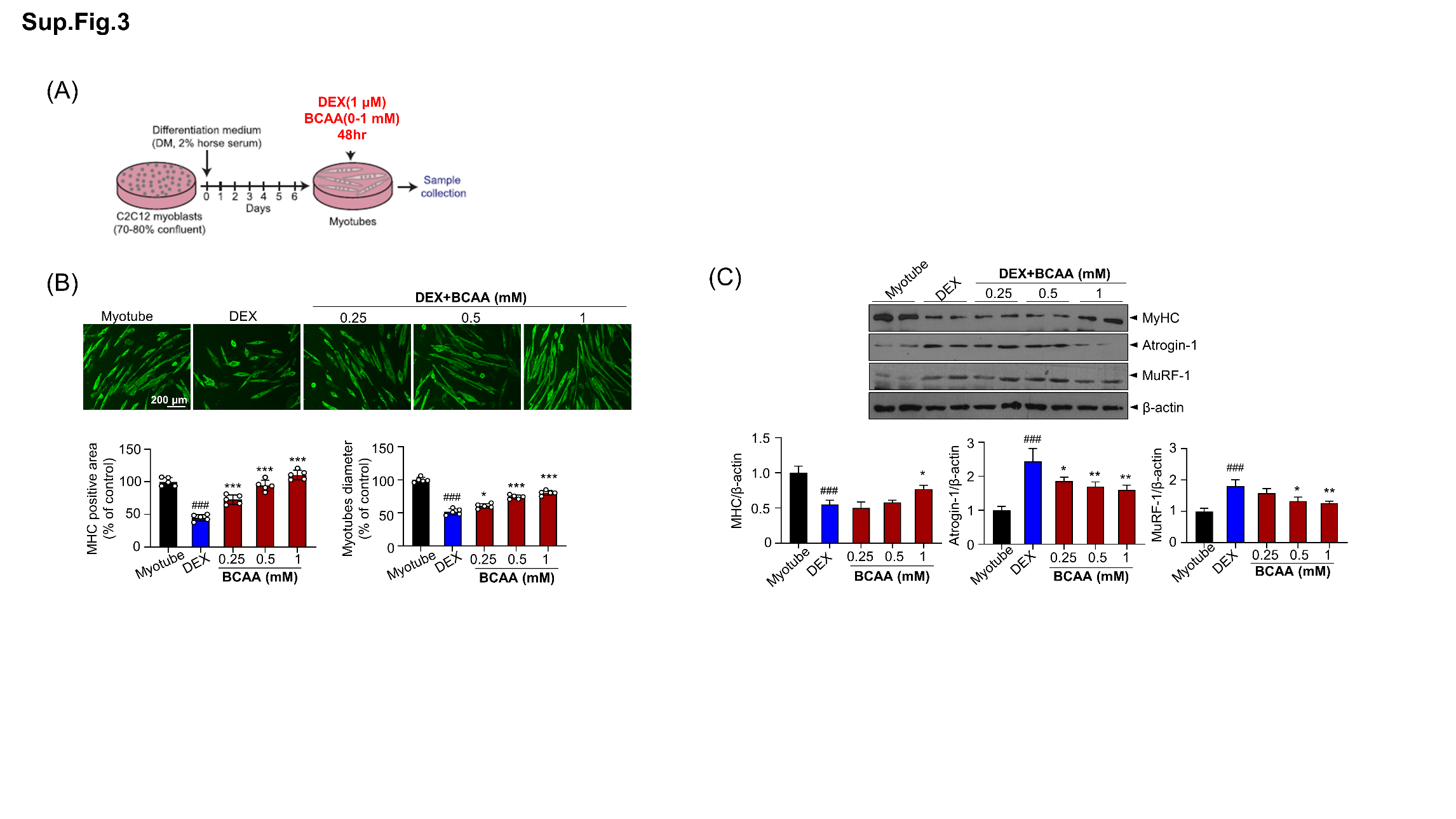


**Supplementary Figure. 3 BCAA attenuates dexamethasone-induced muscle atrophy and enhances myogenesis in C2C12 Cells.** (A) C2C12 myoblasts were cultured to 70–80% confluence, differentiated in DM for 6 days, and then treated with dexamethasone (10 μM) and BCAA (0–1 mM) for 24 h. (B) Myosin heavy chain (MHC) immunofluorescence staining was performed to evaluate myotube morphology. Quantification of MHC-positive area and myotube diameter is shown. (C) Western blotting was conducted to assess MHC, Atrogin-1 and MuRF-1 protein levels, with densitometric analysis. Data are shown as mean ± SD (n=5 (B), n=3 (C), animals/group). ( ^#^*p* < 0.05, ^##^*p* < 0.01, ^###^*p* < 0.001 vs Myotube ; ^*^*p* < 0.05, ^**^*p* < 0.01, ^***^*p* < 0.001 vs. dexamethasone).


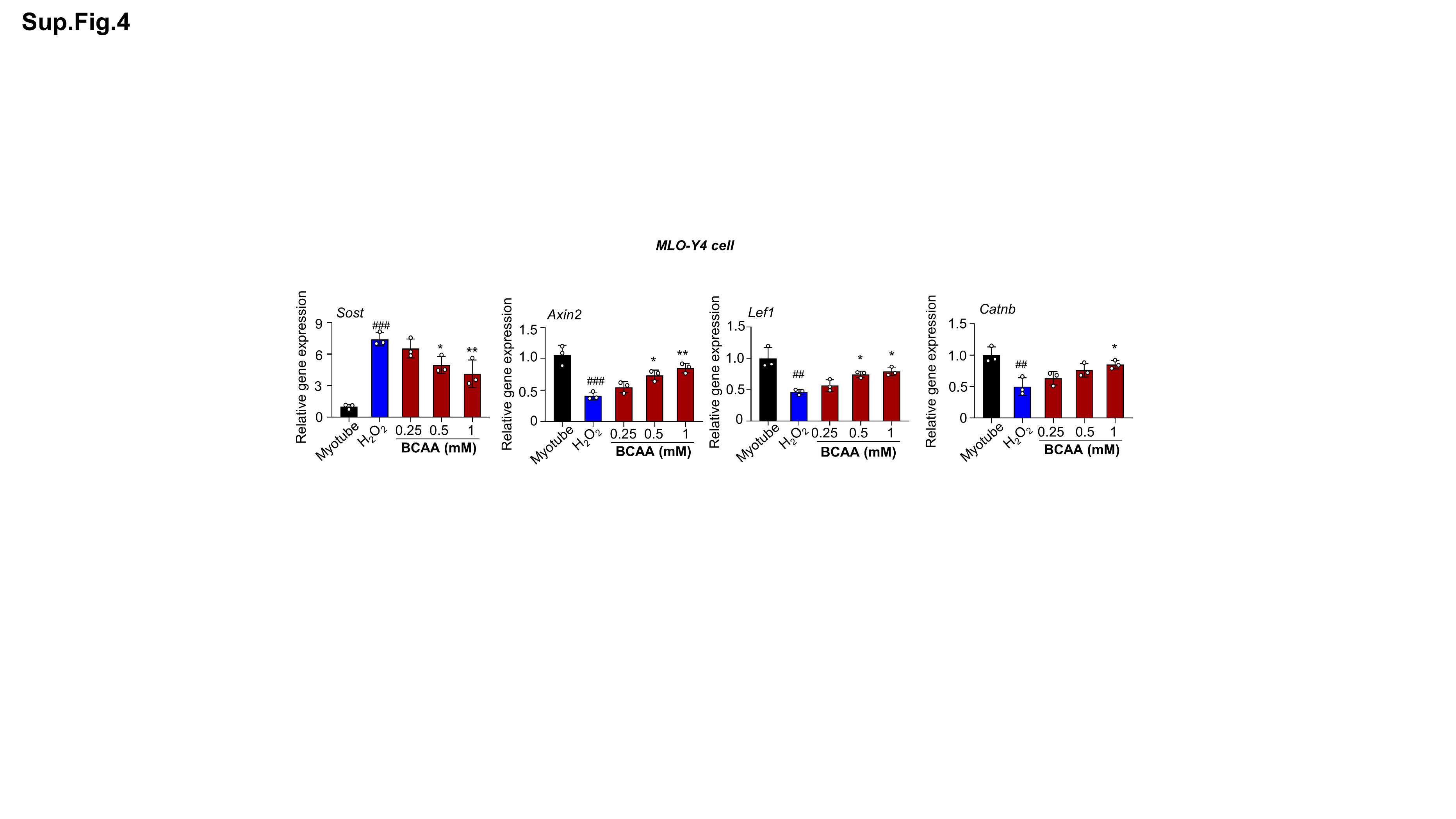


**Supplementary Figure. 4 BCAA modulates sclerostin and Wnt pathway gene expression under oxidative stress.** mRNA levels of *Sost, Axin2, Lef1*, and *Catnb* measured in MLO-Y4 osteocytic cells after H₂O₂ exposure with or without BCAA treatment using qRT-PCR. Data are shown as mean ± SD. (n=3, ^#^*p* < 0.05, ^##^*p* < 0.01, ^###^*p* < 0.001 vs Myotube ; ^*^*p* < 0.05, ^**^*p* < 0.01, ^***^*p* < 0.001 vs. H₂O₂).
